# Effect of pre-exposure use of hydroxychloroquine on COVID-19 mortality: a population-based cohort study in patients with rheumatoid arthritis or systemic lupus erythematosus using the OpenSAFELY platform

**DOI:** 10.1016/S2665-9913(20)30378-7

**Published:** 2020-11-05

**Authors:** Christopher T Rentsch, Nicholas J DeVito, Brian MacKenna, Caroline E Morton, Krishnan Bhaskaran, Jeremy P Brown, Anna Schultze, William J Hulme, Richard Croker, Alex J Walker, Elizabeth J Williamson, Chris Bates, Seb Bacon, Amir Mehrkar, Helen J Curtis, David Evans, Kevin Wing, Peter Inglesby, Rohini Mathur, Henry Drysdale, Angel Y S Wong, Helen I McDonald, Jonathan Cockburn, Harriet Forbes, John Parry, Frank Hester, Sam Harper, Liam Smeeth, Ian J Douglas, William G Dixon, Stephen J W Evans, Laurie Tomlinson, Ben Goldacre

**Affiliations:** a Electronic Health Records Research Group, Faculty of Epidemiology and Population Health, London School of Hygiene & Tropical Medicine, London, UK; b The DataLab, Nuffield Department of Primary Care Health Sciences, University of Oxford, Oxford, UK; c The Phoenix Partnership, Horsforth, Leeds, UK; d Centre for Epidemiology Versus Arthritis, The University of Manchester, Manchester, UK

## Abstract

**Background:**

Hydroxychloroquine has been shown to inhibit entry of severe acute respiratory syndrome coronavirus 2 (SARS-CoV-2) into epithelial cells in vitro, but clinical studies found no evidence of reduced mortality when treating patients with COVID-19. We aimed to evaluate the effectiveness of hydroxychloroquine for prevention of COVID-19 mortality, as opposed to treatment for the disease.

**Methods:**

We did a prespecified observational, population-based cohort study using national primary care data and linked death registrations in the OpenSAFELY platform, which covers approximately 40% of the general population in England, UK. We included all adults aged 18 years and older registered with a general practice for 1 year or more on March 1, 2020. We used Cox regression to estimate the association between ongoing routine hydroxychloroquine use before the COVID-19 outbreak in England (considered as March 1, 2020) compared with non-users of hydroxychloroquine and risk of COVID-19 mortality among people with rheumatoid arthritis or systemic lupus erythematosus. Model adjustment was informed by a directed acyclic graph.

**Findings:**

Between Sept 1, 2019, and March 1, 2020, of 194 637 people with rheumatoid arthritis or systemic lupus erythematosus, 30 569 (15·7%) received two or more prescriptions of hydroxychloroquine. Between March 1 and July 13, 2020, there were 547 COVID-19 deaths, 70 among hydroxychloroquine users. Estimated standardised cumulative COVID-19 mortality was 0·23% (95% CI 0·18 to 0·29) among users and 0·22% (0·20 to 0·25) among non-users; an absolute difference of 0·008% (−0·051 to 0·066). After accounting for age, sex, ethnicity, use of other immunosuppressive drugs, and geographical region, no association with COVID-19 mortality was observed (HR 1·03, 95% CI 0·80 to 1·33). We found no evidence of interactions with age or other immunosuppressive drugs. Quantitative bias analyses indicated that our observed associations were robust to missing information for additional biologic treatments for rheumatological disease. We observed similar associations with the negative control outcome of non-COVID-19 mortality.

**Interpretation:**

We found no evidence of a difference in COVID-19 mortality among people who received hydroxychloroquine for treatment of rheumatological disease before the COVID-19 outbreak in England. Therefore, completion of randomised trials investigating pre-exposure prophylactic use of hydroxychloroquine for prevention of severe outcomes from COVID-19 are warranted.

**Funding:**

Medical Research Council.

## Introduction

Hydroxychloroquine, a commonly used conventional synthetic disease-modifying antirheumatic drug (DMARD), is indicated for treatment of rheumatoid arthritis and systemic lupus erythematosus.^1^ Early in the severe acute respiratory syndrome coronavirus 2 (SARS-CoV-2) pandemic, it was suggested that hydroxychloroquine might have benefits for the treatment and prevention of COVID-19.^2^, ^3^ Hydroxychloroquine has since been investigated in several clinical trials^4^, ^5^, ^6^, ^7^, ^8^, ^9^ and observational cohorts^10^, ^11^, ^12^ with no evidence of therapeutic efficacy in the treatment of patients who are admitted to hospital with symptomatic COVID-19.

Evaluations of the effectiveness of pre-exposure or post-exposure prophylactic use of hydroxychloroquine for prevention, as opposed to treatment, of SARS-CoV-2 infection or severe COVID-19 outcomes are scarce.^13^ One randomised, controlled trial examining hydroxychloroquine as post-exposure prophylaxis did not show significant benefit in preventing SARS-CoV-2 infection, although uncertainty in results could not exclude a possible benefit.^14^ Other trials of pre-exposure or post-exposure use of hydroxychloroquine for the prevention of severe COVID-19 outcomes are ongoing.^4^ In this large, population-based cohort study, we aimed to investigate whether ongoing routine hydroxychloroquine use in the 6 months before March 1, 2020, considered as the start of the outbreak in England, UK (ie, analogous to pre-exposure prophylaxis) was associated with lower risk of COVID-19 mortality.

**Research in context**

**Evidence before this study**

Published trials and observational studies to date have shown no evidence of a benefit of hydroxychloroquine as a treatment for patients admitted to hospital who already have COVID-19. A separate question remains as to whether routine ongoing use of hydroxychloroquine in people without COVID-19 protects against new infections or severe outcomes. We searched MEDLINE and PubMed on Sept 3, 2020, for pharmaco-epidemiological studies evaluating hydroxychloroquine for prevention of severe COVID-19 outcomes. The keywords “hydroxychloroquine AND (COVID OR coronavirus OR SARS-CoV-2) AND (prophyl* OR prevent*) AND (rate OR hazard OR odds OR risk)” were used and results were filtered to articles published since Sept 3, 2019, in English, with abstracts available. 109 papers were identified for screening; none investigated pre-exposure prophylactic use of hydroxychloroquine for prevention of severe COVID-19 outcomes. Clinical trials of prophylactic use of hydroxychloroquine are ongoing; however, the largest trial does not expect to meet recruitment targets due to “unjustified extrapolation and exaggerated safety concerns together with intense politicisation and negative publicity.” In the absence of reported clinical trials, evidence can be generated from real-world data to support the need for randomised clinical trials.

**Added value of this study**

In this population-based cohort study, which uses a database that includes around 40% of the population of England, UK, we investigated whether routine use of hydroxychloroquine before the COVID-19 outbreak (ie, pre-exposure prophylaxis) prevented COVID-19 mortality. With use of robust pharmaco-epidemiological methods, we found no evidence to support a substantial benefit of hydroxychloroquine in preventing COVID-19 mortality. At the same time, we have shown no evidence of harm. The absence of clear harms or benefits supports clinical equipoise to justify continuing randomised trials. We have shown in this study that it is feasible to address specific hypotheses about medicines in a rapid and transparent manner to inform interim clinical decision making and support the need for large-scale, randomised trial data.

**Implications of all the available evidence**

This is the first study, to our knowledge, to investigate the ongoing routine use of hydroxychloroquine and risk of COVID-19 mortality in a general population. Although we found no evidence of any protective benefit, due to the observational nature of the study, residual confounding remains a possibility. Completion of trials for prevention of severe outcomes is warranted, but before the completion of these, we found no evidence to support the use of hydroxychloroquine for prevention of COVID-19 mortality.

## Methods

### Study design and participants

We did an observational cohort study using electronic health record data from primary care practices using The Phoenix Partnership software linked to the UK's Office for National Statistics death registrations through OpenSAFELY. OpenSAFELY is a data analytics platform developed during the COVID-19 pandemic to allow near real-time analysis of pseudonymised primary care records at scale, covering approximately 40% of the population in England, operating within the electronic health record vendor's highly secure data centre.^15^, ^16^ Pseudonymised structured data include demographics, medications prescribed from primary care, diagnoses, and laboratory measures. Details on information governance of the OpenSAFELY platform can be found in the appendix (p 2)).

We included all adults aged 18 years and older registered with a general practice for 1 year or more on March 1, 2020 (index date), with information on age, sex, and deprivation. Within this source population, we identified people with one or more Read diagnostic codes^17^ for rheumatoid arthritis or systemic lupus erythematosus 6 months or more before the index date and who therefore had an indication for hydroxychloroquine use. Detailed information on the Read codes used for rheumatoid arthritis and systemic lupus erythematosus is available online. We studied people with these conditions to minimise the potential for confounding by indication when estimating the effectiveness of hydroxychloroquine use rather than investigating how to prevent severe COVID-19 in this population.

This study was approved by the Health Research Authority (REC 20/LO/0651) and by the London School of Hygiene & Tropical Medicine Ethics Board (number 21863). Participant consent was not required.

### Procedures

The exposure of interest was regular use of hydroxychloroquine (≥2 prescriptions in the 6 months before the index date, termed users) compared with no regular use of hydroxychloroquine regardless of any other medication use (termed non-users). The primary outcome was COVID-19 mortality, defined by the presence of International Classification of Diseases-10 codes U07.1 (COVID-19, virus identified) or U07.2 (COVID-19, virus not identified) on the death certificate.^18^ We followed-up participants from index date until date of death, or 7 days before the last date of availability of Office for National Statistics mortality data to account for reporting lag, whichever came first. People not exposed to hydroxychloroquine before the index date were censored if prescribed it during follow-up (<2% of non-users).

Potential determinants of regular hydroxychloroquine use and COVID-19 mortality were identified by reviewing existing literature and through discussions with clinicians. As this research was a study of prevalent hydroxychloroquine users, we included determinants that might have influenced the initial choice of treatment and whether people remained on treatment. The full list of prespecified variables comprised age, sex, ethnicity, index of multiple deprivation quintile (derived from the individual's postcode at lower super output area level for a high degree of precision), other immunosuppressive drugs (ie, other conventional synthetic DMARDs, oral corticosteroids), smoking status, prescribed non-steroidal anti-inflammatory drugs (NSAIDs), body-mass index, hypertension, diabetes severity as measured by diagnostic codes and glycated haemoglobin (HbA_1c_), heart disease, liver disease, respiratory disease excluding asthma, kidney disease as measured by estimated glomerular filtration rate, stroke, dementia, cancer, and influenza vaccination in the 2019–20 season. Our methods for creating codelists have been previously described;^16^ these methods included clinical and epidemiological review and sign-off by at least two authors. Detailed information on codelists is available online. We developed a directed acyclic graph (DAG) to identify the minimal set of covariates to adjust for the hypothesised confounding structure, which included age, sex, ethnicity, geographical region, and other immunosuppressive drugs (appendix p 3).

### Statistical analysis

Covariates were summarised with use of descriptive statistics, stratified by exposure status. We used Cox regression models with days since the index date as the timescale to estimate hazard ratios (HRs) and 95% CIs for the association between regular hydroxychloroquine use and COVID-19 mortality. The competing risk of death from causes other than COVID-19 was accounted for by censoring non-COVID-19 deaths, so our analysis therefore estimated cause-specific hazards;^19^ this method reflected our interest in establishing the effect of hydroxychloroquine use on COVID-19 mortality as opposed to describing patterns of COVID-19 mortality that might have been driven by the influence of hydroxychloroquine use on competing events. We sequentially adjusted for sex and age using restricted cubic splines; for the minimal adjustment set informed by the DAG; and finally extended for all extracted covariates. Models were stratified by an indicator variable denoting patient population (ie, rheumatoid arthritis or systemic lupus erythematosus) and geographical region. Multiple imputation (ten imputations) was used to account for missing ethnicity for 23% of the sample, with the imputation model including all extracted covariates and an indicator for the outcome. Those with missing body-mass index data were assumed to be non-obese, and those with missing smoking data were assumed to be never-smokers (on the basis of the assumption that clinicians would have recorded details of obesity or smoking if relevant); we did not use multiple imputation for these variables as they are expected to be missing not at random in UK primary care.^20^ Proportional hazards were checked by examining the Schoenfeld residuals over time.

We generated cumulative mortality curves standardised to adjust for different covariate distributions in the exposed group. First, a flexible parametric Royston-Parmar model with the same covariates as the DAG-informed Cox model was fitted, with the baseline hazard modelled with use of a three degrees-of-freedom spline. The survival function was predicted from this model for every individual with regular hydroxychloroquine use and averaged (ie, mean taken at each day of analysis time) to produce the curve for the exposed group. To produce the standardised comparison curve, the survival functions were predicted and averaged again for the same individuals, but with exposure set to zero. Patient population was included in the flexible parametric model as a binary indicator variable because the model could not converge with both patient population and geographical region as stratification variables. Comparisons between Cox and Royston-Parmar models can be found in the appendix (p 13)).

We evaluated prespecified interactions to establish whether the association between regular hydroxychloroquine use and COVID-19 mortality varied by age, exposure to other conventional synthetic DMARDs, oral corticosteroids, and NSAIDs. Two-sided p values were calculated from Wald tests on interaction terms.

We adjusted for ethnicity in a model excluding people with missing ethnicity and compared results with those from multiple imputation. In primary analyses, less than 2% of the comparison group had one prescription of hydroxychloroquine in the 6-month exposure window. We redefined exposure as one or more prescriptions in the 3 months before the index date. We compared results from primary models stratified for patient population (rheumatoid arthritis or systemic lupus erythematosus) to a model that included patient population as a binary indicator variable as well as modelling each population separately.

We calculated bias-adjusted HRs to evaluate how adjustment for biologic DMARDs, including targeted synthetic DMARDs, which were not available for this analysis, might have produced different results under differing assumptions of prevalence and effect on COVID-19 mortality.^21^ Prevalence of biologic DMARDs in each exposure group was estimated from pre-existing scientific literature (18% among users and 21% among non-users);^22^ however, we also examined more extreme values of prevalence (appendix pp 11–12). We assumed a range of potential associations between biologic DMARDs and COVID-19 mortality from 0·8 to 1·2.

Our analyses used non-COVID-19 mortality as a negative control outcome, censoring at COVID-19 death. We hypothesised that if associations observed in primary analyses were due to confounding by indication, we would observe a similar association with non-COVID-19 mortality.

We used Python, version 3.8 and Structured Query Language Server 2016, Enterprise SP2 for data management, and Stata, version 16.1 for analysis. All code for data management and analyses in addition to all iterations of the prespecified protocol are archived with version control.

### Role of the funding source

The funders of the study had no role in study design, data collection, data analysis, data interpretation, or writing of the report. CTR, CEM, CB, AJW, and JC had full access to all of the data and the corresponding author had final responsibility for the decision to submit for publication.

## Results

**Figure 1:**
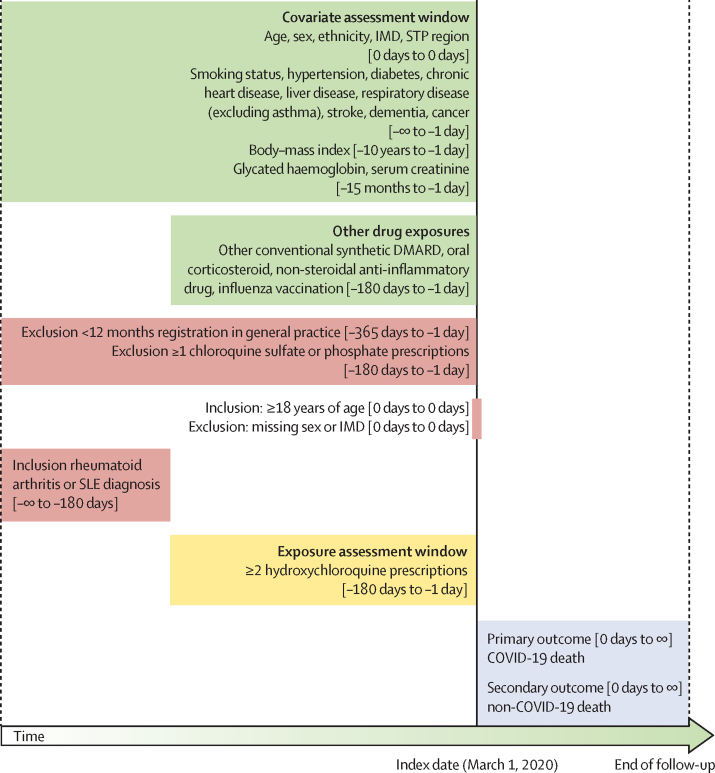
Study diagram End of follow-up was the date of death or 7 days before last date of the Office of National Statistics mortality data to account for reporting lag, or date of first hydroxychloroquine prescription on or after index date (for people without hydroxychloroquine use at index date), whichever came first. STP=Sustainability and Transformation Partnership. DMARD=disease-modifying antirheumatic drug. IMD=index of multiple deprivation. SLE=systemic lupus erythematosus.

**Figure 2:**
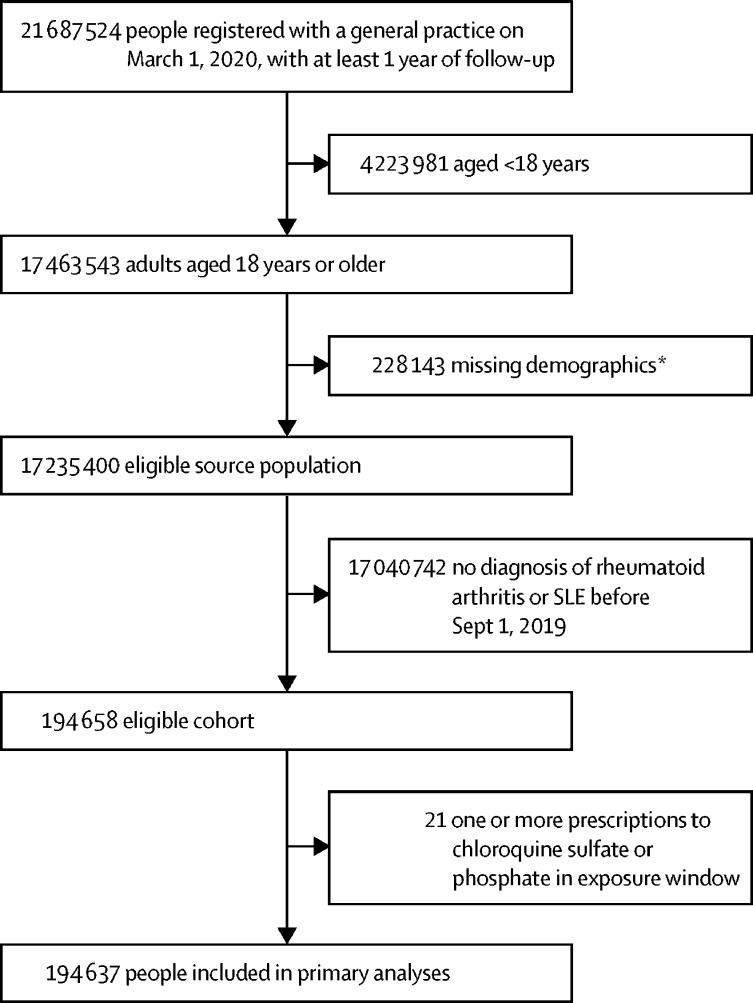
Study profile SLE=systemic lupus erythematosus. *Including sex and index of multiple deprivation.

The study design is shown in figure 1. We identified 194 637 people who were first diagnosed with rheumatoid arthritis or systemic lupus erythematosus at least 6 months before March 1, 2020 (ie, index date), for analysis (figure 2). Of these, 30 569 (15·7%) individuals received two or more prescriptions of hydroxychloroquine in the 6 months before the index date (median number of prescriptions 5, IQR 3–6), showing regular use. Although exposure was ascertained in the 6 months before the index date, 29 707 (97·2%) users received their first hydroxychloroquine prescription before this exposure window and 27 131 (88·8%) initiated 1 or more years before. Hydroxychloroquine users were younger (median age 63 years [IQR 53–72] for users, 66 years [55–76] for non-users) and more likely to be women (76·3% of users were women; 70·2% of non-users were women); other demographic characteristics between exposure groups were broadly similar (table). Hydroxychloroquine users were more likely to be taking other conventional synthetic DMARDs (51·5% *vs* 34·0%), oral corticosteroids (22·5% *vs* 16·3%), and NSAIDs (21·8% *vs* 16·3%) than non-users. Distributions of characteristics in rheumatoid arthritis and systemic lupus erythematosus populations are shown in the appendix (pp 5–8).

**Table:** Baseline characteristics

		**No hydroxychloroquine (n=164 068)**	**Hydroxychloroquine (n=30 569)**	**Total (n=194 637)**
Rheumatoid arthritis		144 151 (87·9%)	23 723 (77·6%)	167 874 (86·2%)
Systemic lupus erythematosus		19 917 (12·1%)	6846 (22·4%)	26 763 (13·8%)
Age, years				
	Median (IQR)	66 (55–76)	63 (53–72)	66 (54–75)
	18–39	11 433 (7·0%)	2276 (7·4%)	13 709 (7·0%)
	40–49	15 829 (9·6%)	3609 (11·8%)	19 438 (10·0%)
	50–59	30 457 (18·6%)	6629 (21·7%)	37 086 (19·1%)
	60–69	37 726 (23·0%)	7973 (26·1%)	45 699 (23·5%)
	70–79	42 090 (25·7%)	7148 (23·4%)	49 238 (25·3%)
	≥80	26 533 (16·2%)	2934 (9·6%)	29 467 (15·1%)
Sex				
	Women	115 106 (70·2%)	23 334 (76·3%)	138 440 (71·1%)
	Men	48 962 (29·8%)	7235 (23·7%)	56 197 (28·9%)
Ethnicity				
	White	112 367 (68·5%)	20 330 (66·5%)	132 697 (68·2%)
	South Asian	8502 (5·2%)	1996 (6·5%)	10 498 (5·4%)
	Black	2425 (1·5%)	572 (1·9%)	2997 (1·5%)
	Mixed	1005 (0·6%)	274 (0·9%)	1279 (0·7%)
	Other	1508 (0·9%)	330 (1·1%)	1838 (0·9%)
	Missing	38 261 (23·3%)	7067 (23·1%)	45 328 (23·3%)
Index of multiple deprivation				
	1 (least deprived)	32 954 (20·1%)	6014 (19·7%)	38 968 (20·0%)
	2	33 351 (20·3%)	6086 (19·9%)	39 437 (20·3%)
	3	32 800 (20·0%)	6142 (20·1%)	38 942 (20·0%)
	4	32 402 (19·7%)	6075 (19·9%)	38 477 (19·8%)
	5 (most deprived)	32 561 (19·8%)	6252 (20·5%)	38 813 (19·9%)
Residence type				
	Rural	38 305 (23·3%)	7351 (24·0%)	45 656 (23·5%)
	Urban	125 763 (76·7%)	23 218 (76·0%)	148 981 (76·5%)
Body-mass index, kg/m^2^				
	<18·5	3692 (2·3%)	680 (2·2%)	4372 (2·2%)
	18·5–24·9	48 051 (29·3%)	8930 (29·2%)	56 981 (29·3%)
	25·0–29·9	52 667 (32·1%)	9203 (30·1%)	61 870 (31·8%)
	30·0–34·9	29 652 (18·1%)	5663 (18·5%)	35 315 (18·1%)
	35·0–39·9	12 372 (7·5%)	2627 (8·6%)	14 999 (7·7%)
	≥40·0	6156 (3·8%)	1571 (5·1%)	7727 (4·0%)
	Missing	11 478 (7·0%)	1895 (6·2%)	13 373 (6·9%)
Smoking status				
	Never	62 705 (38·2%)	11 479 (37·6%)	74 184 (38·1%)
	Former	77 740 (47·4%)	14 692 (48·1%)	92 432 (47·5%)
	Current	23 079 (14·1%)	4332 (14·2%)	27 411 (14·1%)
	Missing	544 (0·3%)	66 (0·2%)	610 (0·3%)
Diabetes				
	No diabetes	133 954 (81·6%)	25 876 (84·6%)	159 830 (82·1%)
	Diabetes, HbA_1c_<7·5%	19 560 (11·9%)	3153 (10·3%)	22 713 (11·7%)
	Diabetes, HbA_1c_ ≥7·5%	7930 (4·8%)	1068 (3·5%)	8998 (4·6%)
	Diabetes, missing HbA_1c_	2624 (1·6%)	472 (1·5%)	3096 (1·6%)
eGFR, mL/min per 1·73m^2^				
	≥60	109 606 (66·8%)	23 765 (77·7%)	133 371 (68·5%)
	30–59	21 153 (12·9%)	3375 (11·0%)	24 528 (12·6%)
	<30	1698 (1·0%)	246 (0·8%)	1944 (1·0%)
	Missing	31 611 (19·3%)	3183 (10·4%)	34 794 (17·9%)
Heart disease		26 292 (16·0%)	4317 (14·1%)	30 609 (15·7%)
Liver disease		2227 (1·4%)	491 (1·6%)	2718 (1·4%)
Respiratory disease (excluding asthma)		22 159 (13·5%)	4521 (14·8%)	26 680 (13·7%)
Neurological condition		11 003 (6·7%)	1715 (5·6%)	12 718 (6·5%)
Hypertension		71 117 (43·3%)	12 287 (40·2%)	83 404 (42·9%)
Cancer		17 144 (10·4%)	2884 (9·4%)	20 028 (10·3%)
Immunosuppression		2399 (1·5%)	570 (1·9%)	2969 (1·5%)
Influenza vaccination in the 2019–20 season		101 112 (61·6%)	21 183 (69·3%)	122 295 (62·8%)
Other medications				
	Other conventional synthetic DMARD	55 780 (34·0%)	15 743 (51·5%)	71 523 (36·7%)
	Azithromycin	751 (0·5%)	197 (0·6%)	948 (0·5%)
	Oral corticosteroid	26 792 (16·3%)	6885 (22·5%)	33 677 (17·3%)
	Non-steroidal anti-inflammatory drug	26 686 (16·3%)	6670 (21·8%)	33 356 (17·1%)

Data are n (%), unless specified. HbA_1c_=glycated haemoglobin. eGFR=estimated glomerular filtration rate. DMARD=disease-modifying antirheumatic drug.

**Figure 3:**
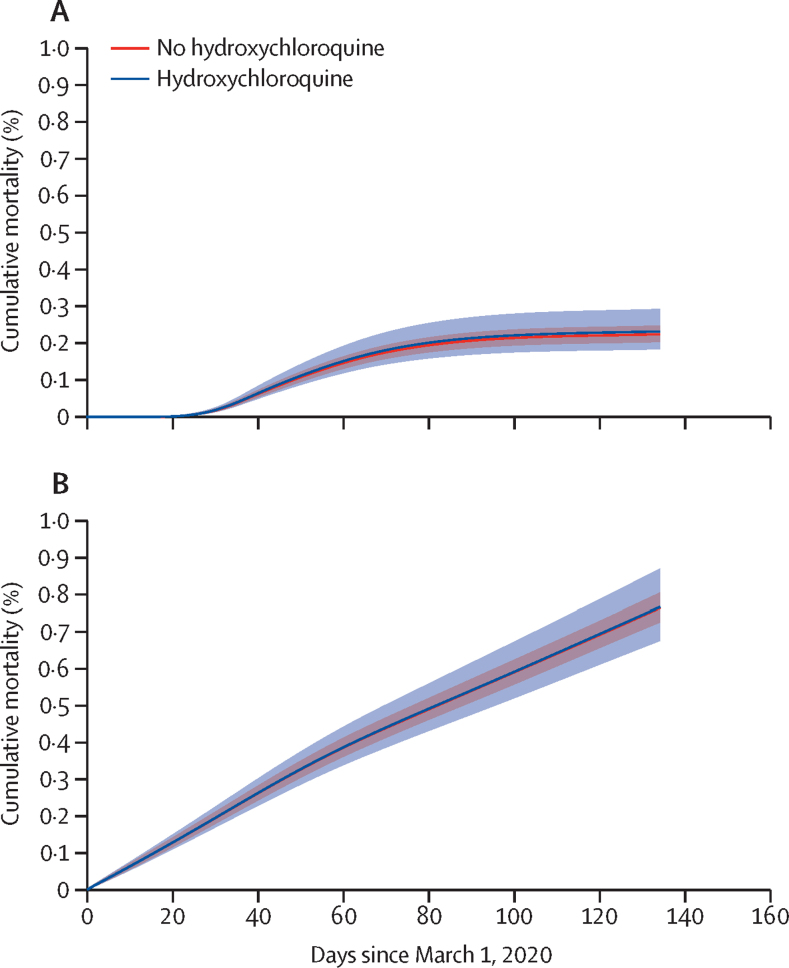
Cumulative mortality by hydroxychloroquine use among people with rheumatoid arthritis or systemic lupus erythematosus (A) Time to COVID-19 death in ONS data and (B) time to non-COVID-19 death in ONS data. Outcome counts were 70 of 547 deaths among hydroxychloroquine users for COVID-19 mortality and 234 of 2003 deaths among hydroxychloroquine users for non-COVID-19 mortality. ONS=Office for National Statistics.

**Figure 4:**
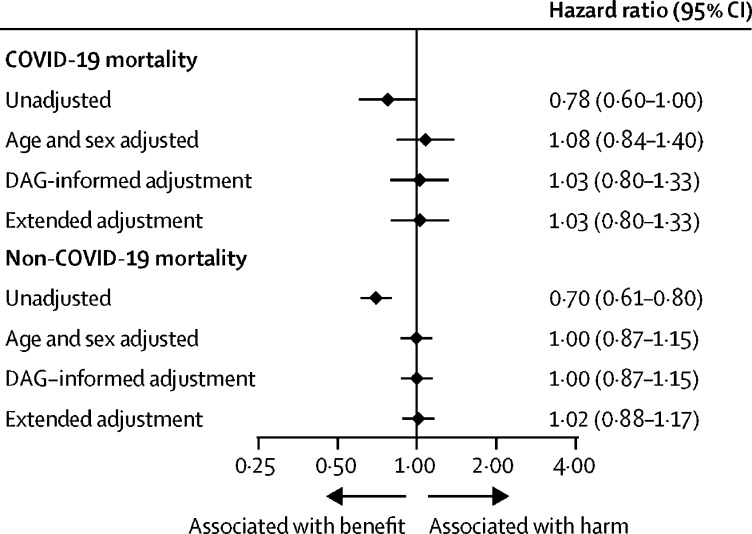
Comparisons between hydroxychloroquine use and no hydroxychloroquine use among people with rheumatoid arthritis or systemic lupus erythematosus Outcome counts were 70 of 547 deaths among hydroxychloroquine users for COVID-19 mortality and 234 of 2003 deaths among hydroxychloroquine users for non-COVID-19 mortality. DAG=directed acyclic graph.

Between March 1 and July 13, 2020, there were 547 COVID-19 deaths among people with rheumatoid arthritis or systemic lupus erythematosus, 70 of which were among regular users of hydroxychloroquine. Estimated standardised cumulative mortality was 0·23% (95% CI 0·18–0·29) among users and 0·22% (0·20–0·25) among non-users at the end of follow-up (figure 3). The absolute cumulative risk difference was 0·008% (95% CI −0·051 to 0·066). In unadjusted analyses, regular users of hydroxychloroquine had a decreased risk of COVID-19 mortality (HR 0·78, 95% CI 0·60–1·00, figure 4). After adjusting for age and sex, there was no longer any evidence of association (HR 1·08, 0·84–1·40). Additionally, adjusting for variables identified in the DAG (HR 1·03, 0·80–1·33) or extending to all covariates (HR 1·03, 0·80–1·33) did not alter conclusions. There was no evidence of interaction by age, exposure to other conventional synthetic DMARDs, oral corticosteroids, or NSAIDs (appendix p 9). When analysing rheumatoid arthritis and systemic lupus erythematosus populations separately, we continued to see no clear effect of hydroxychloroquine use (appendix p 4).

Results from all sensitivity analyses provided similar findings to primary analyses (appendix p 10). In quantitative bias analyses, values of the bias-adjusted association ranged from HR 0·97 (95% CI 0·75–1·26) to HR 1·09 (0·84–1·40; appendix p 12). Hydroxychloroquine use was not associated with the negative control outcome of non-COVID-19 mortality after adjustment for age and sex (HR 1·00, 95% CI 0·87–1·15; Figure 3, Figure 4).

## Discussion

In this national, population-based study of hydroxychloroquine users, we found no evidence that pre-exposure use of hydroxychloroquine was associated with either a beneficial or harmful effect on COVID-19 mortality. The CIs around the relative risk suggest that we could exclude substantial benefit, though a modest benefit or harm on a relative scale could not be ruled out. However, even if hydroxychloroquine provided a benefit, our results showed a maximum absolute risk reduction of 0·05% in the context of an absolute risk of 0·22% of COVID-19 mortality among non-users. If there were substantial differences in health status between the exposure groups, then we would have anticipated to see those differences reflected in analyses of non-COVID-19 mortality. We found no association with this outcome, seen as a negative control. Taken together, our findings do not provide any strong support for a major protective effect from ongoing routine hydroxychloroquine use, as has been previously hypothesised.^2^, ^3^ Our estimates were robust to multiple sensitivity analyses. We showed that it is feasible to address specific hypotheses about medicines in a transparent manner in response to speculation, using OpenSAFELY, and to inform regulatory bodies decision making in the absence of high quality, randomised trial data. However, due to the observational nature of the study, a degree of uncertainty persists that can only be addressed through large-scale randomised trials.

Numerous randomised trials^4^, ^5^, ^6^, ^7^, ^8^, ^9^ have not found any clinical benefit of hydroxychloroquine for treatment, as opposed to prevention, of COVID-19. Previous observational studies had limitations in their design and analysis,^23^ including small sample sizes and focusing only on patients who were admitted to hospital, which might produce spurious associations.^24^ Hydroxychloroquine has been approved for use clinically for more than 50 years, and most studies of hydroxychloroquine for treatment of COVID-19 have not shown substantial harm. However, the RECOVERY trial showed an increased risk of progression to the composite secondary outcome of invasive mechanical ventilation or death in a prespecified secondary analysis.^5^ In addition, the proportion of people with any major cardiac arrhythmia was higher, and concerns about prolongation of the QTc interval have been raised in trials of hydroxychloroquine in combination with azithromycin.^9^

In terms of preventive use of hydroxychloroquine, a randomised trial examining hydroxychloroquine for post-exposure prophylaxis did not show a significant benefit in preventing infection, although the findings were compatible with an absolute risk reduction of as much as 7% in the context of an absolute risk of about 14% in the placebo group.^14^ As we await the reporting of ongoing clinical trials of pre-exposure (as opposed to post-exposure) prophylactic use of hydroxychloroquine, related evidence of drug effectiveness among existing users can be generated from observational data. We found no evidence to support a substantial benefit of hydroxychloroquine in preventing COVID-19 mortality. At the same time, we have shown no significant harm. These findings suggest justification to continue trials of hydroxychloroquine for prevention of COVID-19 to confirm our findings from observational data.

The greatest strength of this study was the large source population with detailed longitudinal, routinely collected data, which enabled us to examine drug effects on an infrequent outcome in a disease-specific cohort. We were able to focus analyses on people with indications for the use of hydroxychloroquine, a key component to mitigate confounding by indication in pharmaco-epidemiological research of real-world data. Before starting the analysis, we developed a DAG to identify a minimal set of covariates to adjust for the hypothesised confounding structure. We also fitted models adjusting for additional characteristics that had been suggested as potentially important when in consultation with clinicians. We did informative sensitivity analyses including quantitative bias analyses to test key assumptions about missing data for biologic DMARD treatments. Lastly, our population included regular users of hydroxychloroquine, who were prescribed doses routinely used in clinical practice, with clarity that hydroxychloroquine administration occurred before exposure to SARS-CoV-2. The optimal timing^25^ and dose^26^ of hydroxychloroquine for therapeutic and prophylactic use for COVID-19 has been debated. The standard dose of hydroxychloroquine in clinical use in England is 200 mg per day or 400 mg per day, commenced without loading dose. This dose is similar to the 200 mg per day used in the COPCOV trial (NCT04303507). A majority (97·2%) of hydroxychloroquine users in our cohort received their first prescription before this exposure window (88·8% initiated ≥1 year before), so we would anticipate that the vast majority of included people were at drug dosages similar or higher than those in clinical trials. Finally, we prespecified our study protocol and have shared all analytical code.

We also recognise possible limitations. One limitation is the risk of residual confounding by use of medications not prescribed in general practice, namely biologic DMARDs. Although most prescriptions in England are supplied by general practitioners in primary care, some medicines, including biologics, might be supplied by hospitals for various reasons, including cost. We have advocated for these data to be more widely shared but, at present, they are not available.^27^ Although nationwide information on the prevalence of concomitant use of biologic DMARDs and hydroxychloroquine over time were unavailable, we showed that our results were robust to a wide range of plausible assumptions about the use of these drugs and their potential relationship with COVID-19 mortality in quantitative bias analysis. A further source of confounding is potential misclassification of rheumatological disease from Read codes, particularly among those not receiving DMARDs who might have less severe disease. In addition, we were unable to capture severity of rheumatological disease. However, the addition of several chronic comorbidities and proxies for health status in extended adjustment did not alter our findings. Another important consideration is the potential for exposure misclassification. As with any observational study that uses prescription data, we were unable to capture whether or how people took their medication. If individuals with prescriptions were taking their medication as directed,^28^ our estimates could potentially be biased toward null findings.^29^ However, we defined exposure based on repeat prescriptions, which is likely to be a good reflection of compliance. We also accounted for people initiating hydroxychloroquine after the index date by censoring observation time at first prescription of hydroxychloroquine (<2% of non-users). In some reportedly rare cases in March, 2020, local shortages of hydroxychloroquine might have occurred due to inappropriate stockpiling; however, the UK has not had major shortages during the COVID-19 outbreak.^30^ People who were more severely immunosuppressed and who had a larger number of comorbidities might have been more likely to shield or adopt stringent physical distancing measures. Although we did not have a direct measure of shielding, there is no evidence to assume it would affect people with rheumatoid arthritis or systemic lupus erythematosus differentially by hydroxychloroquine exposure. In addition, we were able to adjust for oral corticosteroid use, other conventional synthetic DMARDs, and comorbidities in the analysis. Finally, COVID-19 mortality as an outcome reflects the risk of exposure to and acquiring SARS-CoV-2 infection, as well as the risk of developing severe disease and subsequent death. We were not able to explore the risk of SARS-CoV-2 infection in this study due to the absence of complete or representative testing data. However, if hydroxychloroquine had a strong protective effect on the risk of SARS-CoV-2 infection, we would have expected to see this effect reflected in lower risk of COVID-19 mortality.

We studied a large number of people who were prescribed hydroxychloroquine for its licensed purpose and followed them up to look for clear signals of benefit in mortality from COVID-19 and other causes. We found no evidence of benefit after adjusting for important differences in those who had received hydroxychloroquine compared with those who were not prescribed hydroxychloroquine. Completion of randomised trials for prevention of severe outcomes is warranted to support these observational findings. The use of hydroxychloroquine for prevention of COVID-19 mortality outside trial settings is currently not justified.

## Data sharing

All data were linked, stored, and analysed securely within the OpenSAFELY platform. Detailed pseudonymised patient data are potentially re-identifiable and therefore not shared. We rapidly delivered the OpenSAFELY data analysis platform without previous funding to deliver timely analyses of urgent research questions in the context of the global COVID-19 health emergency: now that the platform is established, we are developing a formal process for external users to request access in collaboration with NHS England. Details of this process will be published in the near future on the OpenSAFELY website.
