# Supplementary material for: Effect of pre-exposure use of hydroxychloroquine on COVID-19 mortality: a population-based cohort study in patients with rheumatoid arthritis or systemic lupus erythematosus using the OpenSAFELY platform
Source: Lancet Rheumatol. 2020 Nov 5;3(1):e19–27. doi: 10.1016/S2665-9913(20)30378-7 (PMC7745258; doi:10.1016/S2665-9913(20)30378-7)
Supplement: Supplementary appendix [file mmc1.pdf]

THE LANCET
Rheumatology

Supplementary appendix

This appendix formed part of the original submission and has been peer reviewed. We post it as supplied by the authors.

Supplement to: Rentsch CT, DeVito NJ, MacKenna B, et al. Effect of pre-exposure use of hydroxychloroquine on COVID-19 mortality: a population-based cohort study in patients with rheumatoid arthritis or systemic lupus erythematosus using the OpenSAFELY platform. *Lancet Rheumatol* 2020; published online Nov 5. https://doi.org/10.1016/S2665-9913(20)30378-7.

Supplementary Appendix

Manuscript: Pre-exposure use of hydroxychloroquine for prevention of COVID-19 mortality: a population-based cohort study among people with rheumatoid arthritis or systemic lupus erythematosus using the OpenSAFELY platform

Open source materials: All code for data management and analyses and raw outputs are openly shared online for review and re-use (https://github.com/opensafely/hydroxychloroquine-research). All iterations of the pre-specified study protocol are archived with version control (https://github.com/opensafely/hydroxychloroquine-research/tree/master/protocol).

Table of Contents

Information governance and ethics 2

Patient and public involvement 2

eFigure 1. Directed acyclic graph 3

eFigure 2. Cumulative COVID-19 mortality by population 4

eTable 1. Demographic and clinical characteristics of 167,874 people with rheumatoid arthritis 5

eTable 2. Demographic and clinical characteristics of 26,763 people with systemic lupus erythematosus (SLE) 7

eTable 3. Interactions with HCQ use on the risk of COVID-19 mortality 9

eTable 4. Sensitivity analyses 10

Estimating prevalence of biologic DMARDs by exposure group for eTable 5 11

eTable 5. Bias-adjusted associations using a range of estimated prevalence of biologic DMARDs and associations with COVID-19 mortality 12

eTable 6. Comparison of estimates between stratified Cox proportional hazards models and Royston-Parmar flexible parametric models 13

The RECORD statement – checklist of items, extended from the STROBE statement, that should be reported in observational studies using routinely collected health data 14

References 22

Information governance and ethics

NHS England is the data controller; TPP is the data processor; and the key researchers on OpenSAFELY are acting on behalf of NHS England. OpenSAFELY is hosted within the TPP environment which is accredited to the ISO 27001 information security standard and is NHS IG Toolkit compliant;[1, 2] patient data are pseudonymised for analysis and linkage using industry standard cryptographic hashing techniques; all pseudonymised datasets transmitted for linkage onto OpenSAFELY are encrypted; access to the platform is via a virtual private network (VPN) connection, restricted to a small group of researchers who hold contracts with NHS England and only access the platform to initiate database queries and statistical models. All database activity is logged; only aggregate statistical outputs leave the platform environment following best practice for anonymisation of results such as statistical disclosure control for low cell counts.[3] The OpenSAFELY platform adheres to the data protection principles of the UK Data Protection Act 2018 and the EU General Data Protection Regulation (GDPR) 2016. In March 2020, the Secretary of State for Health and Social Care used powers under the UK Health Service (Control of Patient Information) Regulations 2002 (COPI) to require organisations to process confidential patient information for the purposes of protecting public health, providing healthcare services to the public and monitoring and managing the COVID-19 outbreak and incidents of exposure.[4] Taken together, these provide the legal bases to link patient datasets on the OpenSAFELY platform. This study was approved by the Health Research Authority (REC reference 20/LO/0651) and by the LSHTM Ethics Board (ref 21863).

Patient and public involvement

Patients were not formally involved in developing this specific study design that was developed rapidly in the context of a global health emergency. We have developed a publicly available website https://opensafely.org/ through which we invite any patient or member of the public to contact us regarding this study or the broader OpenSAFELY project.

eFigure 1. Directed acyclic graph

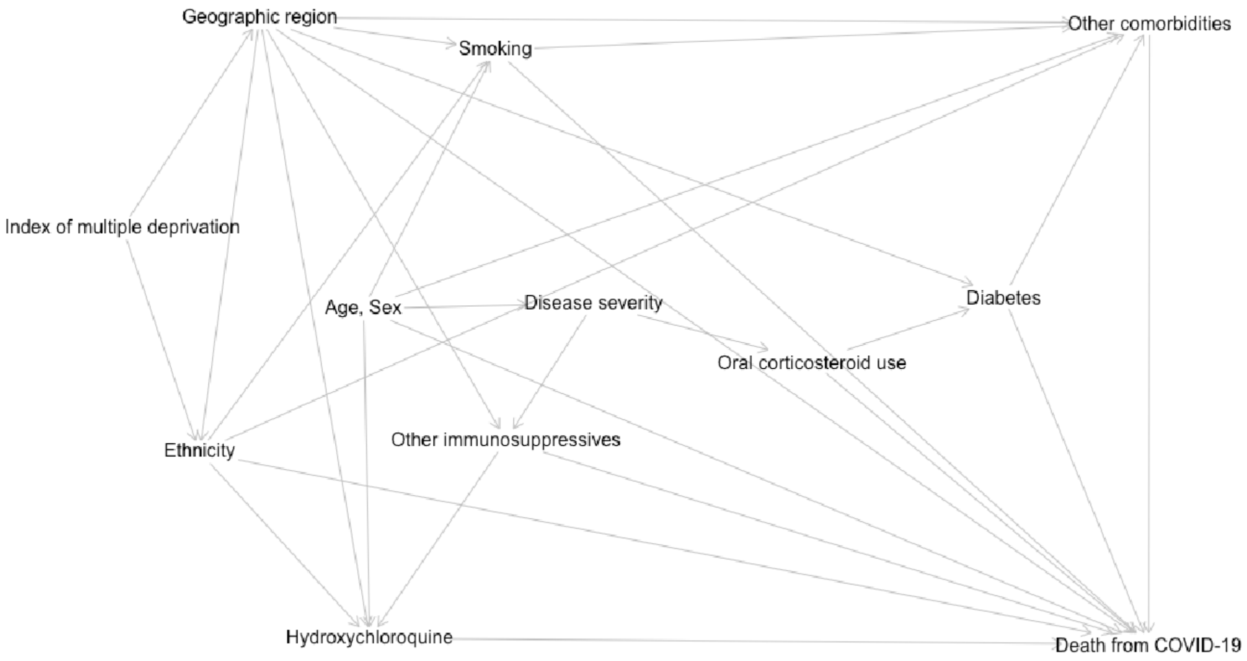

Minimal adjustment set: age, sex, ethnicity, geographic region, other immunosuppressives
Notes: Disease severity referred to that of rheumatoid arthritis or systemic lupus erythematosus. Other immunosuppressives included other synthetic and biologic disease-modifying antirheumatic drugs and oral corticosteroids. Other comorbidities included hypertension, diabetes severity, heart disease, liver disease, respiratory disease excluding asthma, kidney disease, stroke, dementia, cancer

eFigure 2. Cumulative COVID-19 mortality by population

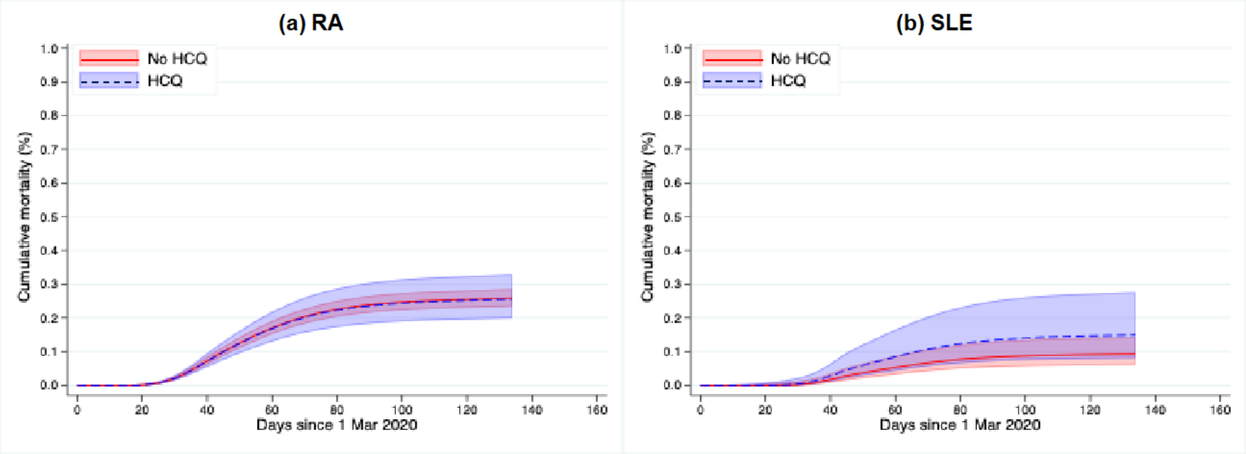

Abbreviations: COVID-19, coronavirus disease 2019; DAG, directed acyclic graph; sDMARD, synthetic disease-modifying antirheumatic drug; STP, Sustainability and Transformation Partnership (a National Health Service administrative region)
Outcome counts: RA population, 60 of 509 deaths among HCQ; SLE population, 10 of 38 deaths among HCQ
Notes: Cumulative mortality predicted using a Royston-Parmar model including all covariates from a DAG-Informed model, adjusted for age, sex, ethnicity, other immunosuppressives (other sDMARDs, oral corticosteroids), and stratified by STP of the patient's general practice. Baseline hazard parametrised as a 3-degrees-of-freedom cubic spline. Predictions standardised to the covariate distribution of the HCQ group.

eTable 1. Demographic and clinical characteristics of 167,874 people with rheumatoid arthritis

	Total	No HCQ	HCQ
Sample size, n	167,874 (100.0)	144,151 (100.0)	23,723 (100.0)
**Demographics**			
Age, years			
Median (IQR)	67 (56-76)	67 (56-76)	65 (55-73)
18-39	10,088 (6.0)	8,957 (6.2)	1,131 (4.8)
40-49	15,111 (9.0)	12,822 (8.9)	2,289 (9.6)
50-59	30,917 (18.4)	26,023 (18.1)	4,894 (20.6)
60-69	40,035 (23.8)	33,464 (23.2)	6,571 (27.7)
70-79	44,491 (26.5)	38,279 (26.6)	6,212 (26.2)
≥80	27,232 (16.2)	24,606 (17.1)	2,626 (11.1)
Sex			
Female	116,406 (69.3)	99,238 (68.8)	17,168 (72.4)
Male	51,468 (30.7)	44,913 (31.2)	6,555 (27.6)
Ethnicity			
White	115,869 (69.0)	99,746 (69.2)	16,123 (68.0)
South Asian	8,511 (5.1)	7,081 (4.9)	1,430 (6.0)
Black	1,981 (1.2)	1,688 (1.2)	293 (1.2)
Mixed	881 (0.5)	724 (0.5)	157 (0.7)
Other	1,354 (0.8)	1,168 (0.8)	186 (0.8)
Missing	39,278 (23.4)	33,744 (23.4)	5,534 (23.3)
Index of multiple deprivation			
1 (least deprived)	33,439 (19.9)	28,787 (20.0)	4,652 (19.6)
2	33,922 (20.2)	29,199 (20.3)	4,723 (19.9)
3	33,702 (20.1)	28,860 (20.0)	4,842 (20.4)
4	33,311 (19.8)	28,614 (19.9)	4,697 (19.8)
5 (most deprived)	33,500 (20.0)	28,691 (19.9)	4,809 (20.3)
Residence type			
Rural	39,577 (23.6)	33,829 (23.5)	5,748 (24.2)
Urban	128,297 (76.4)	110,322 (76.5)	17,975 (75.8)
Body mass index, kg/m^2			
<18.5	3,721 (2.2)	3,205 (2.2)	516 (2.2)
18.5-24.9	48,185 (28.7)	41,443 (28.7)	6,742 (28.4)
25-29.9	54,164 (32.3)	46,823 (32.5)	7,341 (30.9)
30-34.9	31,102 (18.5)	26,617 (18.5)	4,485 (18.9)
35-39.9	13,159 (7.8)	11,103 (7.7)	2,056 (8.7)
≥40	6,778 (4.0)	5,521 (3.8)	1,257 (5.3)
Missing	10,765 (6.4)	9,439 (6.5)	1,326 (5.6)
Smoking status			
Never	63,068 (37.6)	54,640 (37.9)	8,428 (35.5)
Former	81,304 (48.4)	69,376 (48.1)	11,928 (50.3)
Current	23,023 (13.7)	19,696 (13.7)	3,327 (14.0)
Missing	479 (0.3)	439 (0.3)	40 (0.2)
**Clinical conditions**			
Diabetes			
No diabetes	136,690 (81.4)	116,885 (81.1)	19,805 (83.5)
Diabetes, HbA1c <7.5%	20,294 (12.1)	17,659 (12.3)	2,635 (11.1)
Diabetes, HbA1c ≥7.5%	8,171 (4.9)	7,261 (5.0)	910 (3.8)
Diabetes, missing HbA1c	2,719 (1.6)	2,346 (1.6)	373 (1.6)
eGFR, ml/min/1.73m^2			

≥60	117,522 (70.0)	98,630 (68.4)	18,892 (79.6)
30-59	21,998 (13.1)	19,189 (13.3)	2,809 (11.8)
<30	1,622 (1.0)	1,445 (1.0)	177 (0.7)
Missing	26,732 (15.9)	24,887 (17.3)	1,845 (7.8)
Heart disease	27,249 (16.2)	23,708 (16.4)	3,541 (14.9)
Liver disease	2,111 (1.3)	1,787 (1.2)	324 (1.4)
Respiratory disease (excluding asthma)	23,925 (14.3)	20,083 (13.9)	3,842 (16.2)
Neurological condition	10,896 (6.5)	9,646 (6.7)	1,250 (5.3)
Hypertension	73,863 (44.0)	63,955 (44.4)	9,908 (41.8)
Cancer	17,603 (10.5)	15,238 (10.6)	2,365 (10.0)
Immunosuppression	2,200 (1.3)	1,851 (1.3)	349 (1.5)
Influenza vaccination 2019/20	108,883 (64.9)	91,697 (63.6)	17,186 (72.4)
**Other medications**			
Other sDMARD	68,490 (40.8)	54,401 (37.7)	14,089 (59.4)
Azithromycin	850 (0.5)	691 (0.5)	159 (0.7)
Oral corticosteroid	28,858 (17.2)	23,968 (16.6)	4,890 (20.6)
NSAID	30,606 (18.2)	25,042 (17.4)	5,564 (23.5)

Abbreviations: IQR, interquartile range; HbA1c, glycated haemoglobin; eGFR, estimated glomerular filtration rate; sDMARD, synthetic disease-modifying antirheumatic drug; NSAID, non-steroidal anti-inflammatory drug

eTable 2. Demographic and clinical characteristics of 26,763 people with systemic lupus erythematosus (SLE)

	Total	No HCQ	HCQ
Sample size, n	26,763 (100.0)	19,917 (100.0)	6,846 (100.0)
**Demographics**			
Age, years			
Median (IQR)	58 (47-70)	60 (48-71)	55 (44-66)
18-39	3,621 (13.5)	2,476 (12.4)	1,145 (16.7)
40-49	4,327 (16.2)	3,007 (15.1)	1,320 (19.3)
50-59	6,169 (23.1)	4,434 (22.3)	1,735 (25.3)
60-69	5,664 (21.2)	4,262 (21.4)	1,402 (20.5)
70-79	4,747 (17.7)	3,811 (19.1)	936 (13.7)
≥80	2,235 (8.4)	1,927 (9.7)	308 (4.5)
Sex			
Female	22,034 (82.3)	15,868 (79.7)	6,166 (90.1)
Male	4,729 (17.7)	4,049 (20.3)	680 (9.9)
Ethnicity			
White	16,828 (62.9)	12,621 (63.4)	4,207 (61.5)
South Asian	1,987 (7.4)	1,421 (7.1)	566 (8.3)
Black	1,016 (3.8)	737 (3.7)	279 (4.1)
Mixed	398 (1.5)	281 (1.4)	117 (1.7)
Other	484 (1.8)	340 (1.7)	144 (2.1)
Missing	6,050 (22.6)	4,517 (22.7)	1,533 (22.4)
Index of multiple deprivation			
1 (least deprived)	5,529 (20.7)	4,167 (20.9)	1,362 (19.9)
2	5,515 (20.6)	4,152 (20.8)	1,363 (19.9)
3	5,240 (19.6)	3,940 (19.8)	1,300 (19.0)
4	5,166 (19.3)	3,788 (19.0)	1,378 (20.1)
5 (most deprived)	5,313 (19.9)	3,870 (19.4)	1,443 (21.1)
Residence type			
Rural	6,079 (22.7)	4,476 (22.5)	1,603 (23.4)
Urban	20,684 (77.3)	15,441 (77.5)	5,243 (76.6)
Body mass index, kg/m^2			
<18.5	651 (2.4)	487 (2.4)	164 (2.4)
18.5-24.9	8,796 (32.9)	6,608 (33.2)	2,188 (32.0)
25-29.9	7,706 (28.8)	5,844 (29.3)	1,862 (27.2)
30-34.9	4,213 (15.7)	3,035 (15.2)	1,178 (17.2)
35-39.9	1,840 (6.9)	1,269 (6.4)	571 (8.3)
≥40	949 (3.5)	635 (3.2)	314 (4.6)
Missing	2,608 (9.7)	2,039 (10.2)	569 (8.3)
Smoking status			
Never	11,116 (41.5)	8,065 (40.5)	3,051 (44.6)
Former	11,128 (41.6)	8,364 (42.0)	2,764 (40.4)
Current	4,388 (16.4)	3,383 (17.0)	1,005 (14.7)
Missing	131 (0.5)	105 (0.5)	26 (0.4)
**Clinical conditions**			
Diabetes			
No diabetes	23,140 (86.5)	17,069 (85.7)	6,071 (88.7)
Diabetes, HbA1c <7.5%	2,419 (9.0)	1,901 (9.5)	518 (7.6)
Diabetes, HbA1c ≥7.5%	827 (3.1)	669 (3.4)	158 (2.3)
Diabetes, missing HbA1c	377 (1.4)	278 (1.4)	99 (1.4)
eGFR, $ml/min/1.73m^2$			

≥60	15,849 (59.2)	10,976 (55.1)	4,873 (71.2)
30-59	2,530 (9.5)	1,964 (9.9)	566 (8.3)
<30	322 (1.2)	253 (1.3)	69 (1.0)
Missing	8,062 (30.1)	6,724 (33.8)	1,338 (19.5)
Heart disease	3,360 (12.6)	2,584 (13.0)	776 (11.3)
Liver disease	607 (2.3)	440 (2.2)	167 (2.4)
Respiratory disease (excluding asthma)	2,755 (10.3)	2,076 (10.4)	679 (9.9)
Neurological condition	1,822 (6.8)	1,357 (6.8)	465 (6.8)
Hypertension	9,541 (35.6)	7,162 (36.0)	2,379 (34.8)
Cancer	2,425 (9.1)	1,906 (9.6)	519 (7.6)
Immunosuppression	769 (2.9)	548 (2.8)	221 (3.2)
Influenza vaccination 2019/20	13,412 (50.1)	9,415 (47.3)	3,997 (58.4)
**Other medications**			
Other sDMARD	3,033 (11.3)	1,379 (6.9)	1,654 (24.2)
Azithromycin	98 (0.4)	60 (0.3)	38 (0.6)
Oral corticosteroid	4,819 (18.0)	2,824 (14.2)	1,995 (29.1)
NSAID	2,750 (10.3)	1,644 (8.3)	1,106 (16.2)

Abbreviations: IQR, interquartile range; HbA1c, glycated haemoglobin; eGFR, estimated glomerular filtration rate; sDMARD, synthetic disease-modifying antirheumatic drug; NSAID, non-steroidal anti-inflammatory drug

eTable 3. Interactions with HCQ use on the risk of COVID-19 mortality

	No. events	DAG-Informed Adjustment HR	95% CI	p-value
**Age**				
18-59 years				
No HCQ	18 (0.0)	1	(ref)	0.78
HCQ	6 (0.0)	1.50	(0.59-3.79)	
60-69 years				
No HCQ	40 (0.1)	1	(ref)	
HCQ	10 (0.1)	1.17	(0.58-2.35)	
70-79 years				
No HCQ	148 (0.4)	1	(ref)	
HCQ	25 (0.3)	0.97	(0.63-1.48)	
≥80 years				
No HCQ	271 (1.0)	1	(ref)	
HCQ	29 (1.0)	0.92	(0.62-1.35)	
**Other sDMARD**				
No				
No HCQ	311 (0.3)	1	(ref)	0.22
HCQ	41 (0.3)	1.14	(0.82-1.59)	
Yes				
No HCQ	166 (0.3)	1	(ref)	
HCQ	29 (0.2)	0.83	(0.56-1.24)	
**Oral corticosteroid**				
No				
No HCQ	296 (0.2)	1	(ref)	0.97
HCQ	38 (0.2)	1.00	(0.71-1.41)	
Yes				
No HCQ	181 (0.7)	1	(ref)	
HCQ	32 (0.5)	0.99	(0.68-1.45)	
**NSAID**				
No				
No HCQ	443 (0.3)	1	(ref)	0.11
HCQ	62 (0.3)	0.98	(0.75-1.29)	
Yes				
No HCQ	34 (0.1)	1	(ref)	
HCQ	8 (0.1)	1.18	(0.55-2.56)	

Abbreviations: HCQ, hydroxychloroquine; COVID-19, coronavirus disease 2019; DAG, directed acyclic graph; CI, confidence interval; sDMARD, synthetic disease-modifying antirheumatic drug; STP, Sustainability and Transformation Partnership (a National Health Service administrative region)

Notes: DAG-Informed models adjusted for age, sex, ethnicity, other immunosuppressives (other sDMARDs, oral corticosteroids) and stratified by population (i.e., RA or SLE) and STP of the patient's general practice to allow for geographical differences in baseline hazards.

eTable 4. Sensitivity analyses

	No. events	DAG-Informed Adjustment HR	95% CI
**Ethnicity adjustments**			
Full cohort, without adjustment for ethnicity			
No HCQ	477 (0.3)	1	(ref)
HCQ	70 (0.2)	1.03	(0.80-1.33)
Exclude individuals with missing ethnicity			
No HCQ	358 (0.3)	1	(ref)
HCQ	48 (0.2)	0.94	(0.69-1.28)
Additional adjustment for ethnicity			
No HCQ	358 (0.3)	1	(ref)
HCQ	48 (0.2)	0.94	(0.69-1.27)
**Shorten exposure ascertainment to 3 months**			
No HCQ	477 (0.3)	1	(ref)
HCQ	70 (0.2)	1.03	(0.80-1.33)
**Population adjustment**			
Change from stratifying to indicator variable			
No HCQ	477 (0.3)	1	(ref)
HCQ	70 (0.2)	0.94	(0.69-1.27)
**Model populations separately**			
Rheumatoid arthritis (RA)			
No HCQ	449 (0.3)	1	(ref)
HCQ	60 (0.3)	0.98	(0.75-1.29)
Systemic lupus erythematosus (SLE)			
No HCQ	28 (0.1)	1	(ref)
HCQ	10 (0.1)	1.64	(0.68-3.97)

Abbreviations: HCQ, hydroxychloroquine; COVID-19, coronavirus disease 2019; DAG, directed acyclic graph; CI, confidence interval; sDMARD, synthetic disease-modifying antirheumatic drug; STP, Sustainability and Transformation Partnership (a National Health Service administrative region)

Notes: DAG-Informed models adjusted for age, sex, ethnicity, other immunosuppressives (other sDMARDs, oral corticosteroids) and stratified by population (i.e., RA or SLE) and STP of the patient's general practice to allow for geographical differences in baseline hazards.

Estimating prevalence of biologic DMARDs by exposure group for eTable 5

We applied Bayes' rule to obtain estimates of the prevalence of biologic DMARDs by exposure group.

Bayes' rule definition:

$$P(A|B) \ = \frac{P(B|A)\ P(A)}{P(B)}$$

In 2017, among 400,000 rheumatoid arthritis patients in the UK, 84,200 (21%) were treated with biologics.[5] Previous drug utilisation research has shown that 12% of rheumatoid arthritis patients on biologics were co-prescribed hydroxychloroquine.[6] In our cohort of 167,874 people with rheumatoid arthritis, 23,723 (14%) were prescribed hydroxychloroquine.

We substituted biologics for A and hydroxychloroquine use for B in Bayes' rule.

Bayes' rule application:

$$P(B|HCQ) \ = \frac{P(HCQ|B)\ P(B)}{P(HCQ)}$$

$$P(B|HCQ) = \frac{0.12 \ \times \ 0.21}{0.14} \ = 0.18$$

By law of total probability:

$$P(B) = P(B|HCQ)\ P(HCQ) \ + \ P(B|\underline{HCQ})\ P(\underline{HCQ})$$

$$0.21 = 0.18 \times 0.14 \ + \ P(B|\underline{HCQ}) \ \times 0.86$$

$$P(B|\underline{HCQ}) = \frac{0.21 \ - \ (0.18 \ \times \ 0.14)}{0.86} \ = 0.21$$

Given the above assumptions, we estimated prevalence of biologic DMARDs to be 18% among hydroxychloroquine users and 21% among those not prescribed hydroxychloroquine.

We used these estimates to calculate bias-adjusted associations in quantitative bias analyses. To account for uncertainty in these estimates and any variation in prescribing practices between rheumatoid arthritis and systemic lupus erythematosus patient populations, we also examined more extreme values of prevalence (range 3-30%).

eTable 5. Bias-adjusted associations using a range of estimated prevalence of biologic DMARDs and associations with COVID-19 mortality

		Association between bDMARD and COVID-19 mortality			
**Prevalence of bDMARD among exposed**	**Prevalence of bDMARD among unexposed**	**HR 0.80**	**HR 0.90**	**HR 1.10**	**HR 1.20**
18%	21%	1.02 (0.79-1.32)	1.03 (0.79-1.32)	1.03 (0.80-1.33)	1.03 (0.80-1.34)
3%	30%	0.97 (0.75-1.26)	1.00 (0.78-1.29)	1.06 (0.82-1.36)	1.08 (0.84-1.40)
30%	3%	1.09 (0.84-1.40)	1.06 (0.82-1.36)	1.00 (0.78-1.29)	0.98 (0.76-1.26)

Abbreviations: bDMARD, biologic disease-modifying antirheumatic drug; COVID-19, coronavirus disease 2019; HR, hazard ratio

eTable 6. Comparison of estimates between stratified Cox proportional hazards models and Royston-Parmar flexible parametric models

	Stratified Cox (stcox)		Flexible parametric (stpm2)	
	HR	95% CI	HR	95% CI
**COVID-19 mortality**				
HCQ, yes vs. no	1.03	(0.80-1.33)	1.03	(0.80-1.33)
Sex, male vs. female	1.46	(1.23-1.74)	1.45	(1.22-1.72)
Age, spline 1	1.05	(0.98-1.13)	1.05	(0.98-1.13)
Age, spline 2	1.08	(0.97-1.19)	1.08	(0.97-1.19)
Age, spline 3	0.75	(0.50-1.11)	0.74	(0.50-1.10)
Other sDMARD, yes vs. no	0.93	(0.78-1.12)	0.91	(0.76-1.09)
Oral corticosteroid, yes vs. no	2.47	(2.07-2.93)	2.45	(2.06-2.91)
**Non COVID-19 mortality**				
HCQ, yes vs. no	1.00	(0.87-1.15)	1.00	(0.87-1.15)
Sex, male vs. female	1.25	(1.14-1.37)	1.25	(1.14-1.37)
Age, spline 1	1.06	(1.03-1.10)	1.06	(1.03-1.10)
Age, spline 2	1.03	(0.98-1.09)	1.03	(0.98-1.09)
Age, spline 3	0.94	(0.77-1.14)	0.94	(0.77-1.14)
Other sDMARD, yes vs. no	0.68	(0.61-0.75)	0.68	(0.62-0.75)
Oral corticosteroid, yes vs. no	2.03	(1.85-2.23)	2.03	(1.85-2.22)

Note: Cox models (Stata command: stcox) were stratified on STP and RA/SLE population. Royston-Parmar flexible parametric models (Stata command: stpm2) were stratified on STP and included a binary indicator for RA/SLE population as a fixed effect.

The RECORD statement – checklist of items, extended from the STROBE statement, that should be reported in observational studies using routinely collected health data

	Item No.	STROBE items	Location in manuscript where items are reported	RECORD items	Location in manuscript where items are reported
**Title and abstract**					
	1	(a) Indicate the study's design with a commonly used term in the title or the abstract (b) Provide in the abstract an informative and balanced summary of what was done and what was found	(a) Title & Abstract<br><br>(b) Abstract	RECORD 1.1: The type of data used should be specified in the title or abstract. When possible, the name of the databases used should be included.<br><br>RECORD 1.2: If applicable, the geographic region and timeframe within which the study took place should be reported in the title or abstract.<br><br>RECORD 1.3: If linkage between databases was conducted for the study, this should be clearly stated in the title or abstract.	1.1: Abstract – Methods<br><br>1.2: Abstract – Methods & Findings<br><br>1.3: Abstract – Methods
**Introduction**					
Background rationale	2	Explain the scientific background and rationale for the investigation being reported	Background (Para 1)		
Objectives	3	State specific objectives, including any prespecified hypotheses	Background (End of Para 2)		

Methods					
Study Design	4	Present key elements of study design early in the paper	Methods (Study design and population)		
Setting	5	Describe the setting, locations, and relevant dates, including periods of recruitment, exposure, follow-up, and data collection	Methods (Study design and population; Exposure, outcome and follow-up)		
Participants	6	*(a) Cohort study* - Give the eligibility criteria, and the sources and methods of selection of participants. Describe methods of follow-up<br>*Case-control study* - Give the eligibility criteria, and the sources and methods of case ascertainment and control selection. Give the rationale for the choice of cases and controls<br>*Cross-sectional study* - Give the eligibility criteria, and the sources and methods of selection of participants<br><br>*(b) Cohort study* - For matched studies, give matching criteria and number of exposed and unexposed	(a) Methods (Study design and population, para 2)	RECORD 6.1: The methods of study population selection (such as codes or algorithms used to identify subjects) should be listed in detail. If this is not possible, an explanation should be provided.<br><br>RECORD 6.2: Any validation studies of the codes or algorithms used to select the population should be referenced. If validation was conducted for this study and not published elsewhere, detailed methods and results should be provided.<br><br>RECORD 6.3: If the study involved linkage of databases, consider use of a flow diagram or other graphical display to demonstrate the data linkage process, including the number of	6.1: Methods (Study design and population, para 2)<br><br>6.2: Methods: Link to codelist info at: https://codelists.opensafely.org & Covariates<br><br>6.3: Figure 2

		Case-control study - For matched studies, give matching criteria and the number of controls per case		individuals with linked data at each stage.	
Variables	7	Clearly define all outcomes, exposures, predictors, potential confounders, and effect modifiers. Give diagnostic criteria, if applicable.	Methods (Covariates)	RECORD 7.1: A complete list of codes and algorithms used to classify exposures, outcomes, confounders, and effect modifiers should be provided. If these cannot be reported, an explanation should be provided.	7.1: Methods: (Link to codelist info at: https://codelists.opensafely.org & Covariates)
Data sources/ measurement	8	For each variable of interest, give sources of data and details of methods of assessment (measurement). Describe comparability of assessment methods if there is more than one group	Overview in Methods (Exposure, outcome and follow-up; Covariates), detailed information in analytic code.		
Bias	9	Describe any efforts to address potential sources of bias	Methods (statistical methods, sensitivity analysis)		
Study size	10	Explain how the study size was arrived at	Methods (study design an population)		
Quantitative variables	11	Explain how quantitative variables were handled in the analyses. If applicable, describe which groupings were chosen, and why	Methods (statistical methods)		

Statistical methods	12	(a) Describe all statistical methods, including those used to control for confounding<br>(b) Describe any methods used to examine subgroups and interactions<br>(c) Explain how missing data were addressed<br>(d) *Cohort study* - If applicable, explain how loss to follow-up was addressed<br>*Case-control study* - If applicable, explain how matching of cases and controls was addressed<br>*Cross-sectional study* - If applicable, describe analytical methods taking account of sampling strategy<br>(e) Describe any sensitivity analyses	(a-c) Methods (Covariates; Statistical methods, Sensitivity analyses)<br><br>(d) Not applicable for this study<br><br>(e) Methods (Sensitivity analyses)		
Data access and cleaning methods		..		RECORD 12.1: Authors should describe the extent to which the investigators had access to the database population used to create the study population.<br><br>RECORD 12.2: Authors should provide information on the data cleaning methods used in the study.	12.1: Methods (Software and reproducibility; Role of the funding source)<br><br>12.2: Methods (Study design and population; Covariates)

Linkage		..		RECORD 12.3: State whether the study included person-level, institutional-level, or other data linkage across two or more databases. The methods of linkage and methods of linkage quality evaluation should be provided.	Methods (Study design and population)
**Results**					
Participants	13	(a) Report the numbers of individuals at each stage of the study (*e.g.*, numbers potentially eligible, examined for eligibility, confirmed eligible, included in the study, completing follow-up, and analysed)<br>(b) Give reasons for non-participation at each stage.<br>(c) Consider use of a flow diagram	(a-c) Results (Patient characteristics, Figure 1)	RECORD 13.1: Describe in detail the selection of the persons included in the study (*i.e.,* study population selection) including filtering based on data quality, data availability and linkage. The selection of included persons can be described in the text and/or by means of the study flow diagram.	13.1: Results (Patient characteristics, Figure 1)
Descriptive data	14	(a) Give characteristics of study participants (*e.g.*, demographic, clinical, social) and information on exposures and potential confounders<br>(b) Indicate the number of participants with missing data for each variable of interest	(a) Results (Patient characteristics, Table 1, eTables 1 & 2)<br><br>(b) Table 1<br><br>(c) Results (Patient characteristics)		

		(c) *Cohort study* - summarise follow-up time (*e.g.*, average and total amount)			
Outcome data	15	*Cohort study* - Report numbers of outcome events or summary measures over time<br>*Case-control study* - Report numbers in each exposure category, or summary measures of exposure<br>*Cross-sectional study* - Report numbers of outcome events or summary measures	Results (Univariable and multivariable regression)		
Main results	16	(a) Give unadjusted estimates and, if applicable, confounder-adjusted estimates and their precision (e.g., 95% confidence interval). Make clear which confounders were adjusted for and why they were included<br>(b) Report category boundaries when continuous variables were categorized<br>(c) If relevant, consider translating estimates of relative risk into absolute risk for a meaningful time period	(a) Results (Figure 3)<br><br>(b) Results (Table 1)<br><br>(c) Results (Univariable and multivariable regression)		

Other analyses	17	Report other analyses done—e.g., analyses of subgroups and interactions, and sensitivity analyses	Results (Univariable and multivariable regression ; Sensitivity analyses)		
**Discussion**					
Key results	18	Summarise key results with reference to study objectives	Discussion (Key findings)		
Limitations	19	Discuss limitations of the study, taking into account sources of potential bias or imprecision. Discuss both direction and magnitude of any potential bias	Discussion (Strengths and limitations)	RECORD 19.1: Discuss the implications of using data that were not created or collected to answer the specific research question(s). Include discussion of misclassification bias, unmeasured confounding, missing data, and changing eligibility over time, as they pertain to the study being reported.	19.1 Discussion (Strengths and limitations)
Interpretation	20	Give a cautious overall interpretation of results considering objectives, limitations, multiplicity of analyses, results from similar studies, and other relevant evidence	Discussion (Throughout)		
Generalisability	21	Discuss the generalisability (external validity) of the study results	Discussion (Strengths and limitations)		
**Other Information**					

Funding	22	Give the source of funding and the role of the funders for the present study and, if applicable, for the original study on which the present article is based	Funding		
Accessibility of protocol, raw data, and programming code		..		RECORD 22.1: Authors should provide information on how to access any supplemental information such as the study protocol, raw data, or programming code.	Methods (Software and reproducibility)

Reference: Benchimol EI, Smeeth L, Guttmann A, Harron K, Moher D, Petersen I, Sørensen HT, von Elm E, Langan SM, the RECORD Working Committee. The REporting of studies Conducted using Observational Routinely-collected health Data (RECORD) Statement. *PLOS Medicine* 2015; DOI: 10.1371/journal.pmed.1001885

Checklist is protected under Creative Commons Attribution (CC BY) license.

References

1. NHS Digital. Data Security and Protection Toolkit. 2020. Accessed October 6, 2020. [Available from: https://digital.nhs.uk/data-and-information/looking-after-information/data-security-and-information-governance/data-security-and-protection-toolkit.

2. NHS Digital. BETA - Data Security Standards. 2020. Accessed October 6, 2020. [Available from: https://digital.nhs.uk/about-nhs-digital/our-work/nhs-digital-data-and-technology-standards/framework/beta---data-security-standards.

3. NHS Digital. ISB1523: Anonymisation Standard for Publishing Health and Social Care Data. 2020. Accessed October 6, 2020. [Available from: https://digital.nhs.uk/data-and-information/information-standards/information-standards-and-data-collections-including-extractions/publications-and-notifications/standards-and-collections/isb1523-anonymisation-standard-for-publishing-health-and-social-care-data.

4. Secretary of State for Health - UK Government. Coronavirus (COVID-19): notification to organisations to share information. 2020. Accessed October 6, 2020. [Available from: https://www.gov.uk/government/publications/coronavirus-covid-19-notification-of-data-controllers-to-share-information.

5. Kim H. The Roles of Biosimilars in Patient Access to Biologics in UK. Accessed October 6, 2020. [Available from: https://www.england.nhs.uk/expo/wp-content/uploads/sites/18/2018/09/12.30-Improving-patient-care-with-biologics-in-the-management-of-RA-Celltrion-Healthcare-P2C.pdf.

6. Choy E, Taylor P, McAuliffe S, Roberts K, Sargeant I. Variation in the use of biologics in the management of rheumatoid arthritis across the UK. Curr Med Res Opin. 2012;28(10):1733-41.
